# An Initial Survey of the Performances of Exome Variant Analysis and Clinical Reporting Among Diagnostic Laboratories in China

**DOI:** 10.3389/fgene.2020.582637

**Published:** 2020-11-02

**Authors:** Kuo Zhang, Guigao Lin, Dongsheng Han, Yanxi Han, Jian Wang, Yiping Shen, Jinming Li

**Affiliations:** ^1^National Center for Clinical Laboratories, Beijing Hospital, National Center of Gerontology, Institute of Geriatric Medicine, Chinese Academy of Medical Sciences, Beijing, China; ^2^Graduate School, Peking Union Medical College, Chinese Academy of Medical Sciences, Beijing, China; ^3^Department of Medical Genetics and Molecular Diagnostic Laboratory, Shanghai Children’s Medical Center, Shanghai Jiao Tong University School of Medicine, Shanghai, China; ^4^Division of Genetics and Genomics, Boston Children’s Hospital, Boston, MA, United States; ^5^Genetic and Metabolic Central Laboratory, Maternal and Child Health Hospital of Guangxi Zhuang Autonomous Region, Nanning, China

**Keywords:** exome sequencing, variant analysis, variant interpretation, clinical reporting, laboratory performance

## Abstract

Exome sequencing has become an effective diagnostic method for *Mendelian* disorders. But the quality of services differs widely across laboratories in China, particularly in variant classification, even with the adoption of the ACMG guidelines. As an effort of quality control and improvement for better clinical utilization of exome sequencing, we assessed the exome data analysis and clinical reporting among Chinese laboratories. Five raw datasets of real clinical samples with associated phenotypes were sent to 53 laboratories. The participants independently performed secondary analysis, variant classification, and reporting. The first round of results was used for identifying problems associated with these aspects. Subsequently, we implemented several corrective actions and a training program was designed based on the identified issues. A second round of five datasets were sent to the same participants. We compared the performances in variant interpretation and reporting. A total of 85.7% (42/49) of participants correctly identified all the variants related with phenotype. Many lines of evidence using the ACMG guidelines were incorrectly utilized, which resulted in a large inter-laboratory discrepancy. After training, the evidence usage problems significantly improved, leading to a more consistent outcome. Participants improved their exome data analysis and clinical reporting capability. Targeted training and a deeper understanding of the ACMG guidelines helped to improve the clinical exome sequencing service in terms of consistency and accuracy in variant classification in China.

## Introduction

Exome sequencing has proven to be an effective strategy for gene discovery and clinical diagnosis for patients with Mendelian disorders, which herald a new era of genomic medicine (Yang et al., 2013, 2014; Lee et al., 2014; Retterer et al., 2016; Wright et al., 2018). However, genome-wide variant analysis is highly complex and challenging for every clinical laboratory. From processing massive raw data to reporting variants associated with phenotype, the bioinformatics pipeline consists of a series of steps and data transformations using multiple algorithms, software, databases, and operation environment (Roy et al., 2018). There is no standard for how to use these components to analyze the outputs of different sequencing platforms, and for how these tools are combined and used in context for patient care (Brownstein et al., 2014). Many issues have been reported regarding the inconsistency of variant calling, variant annotation, variant filtration, variant classification, and variant reporting in clinical diagnostic settings. For example, a study analyzed the same set of raw sequence data with different alignment and variant-calling pipelines, showing that there was a significant discrepancy in SNV and indel calling across exomes and demonstrating fundamental methodological variation between commonly used tools (O’Rawe et al., 2013). Annotation discrepancy has also been reported when using different transcript sets or software in annotation (McCarthy et al., 2014), splicing variants were the category with the greatest discrepancy (McCarthy et al., 2014; Zhao and Zhang, 2015). To facilitate the clinical implementation of genomic medicine, it is important to obtain a robust, accurate, and consistent variant analysis pipeline.

In terms of variant classification, previous reports revealed extensive discordance between laboratories (Brownstein et al., 2014; Landrum et al., 2016; Pepin et al., 2016). A total of 17% of variants in ClinVar had non-uniform classifications from multiple submitters, demonstrating the need for standardized guidelines for variant interpretation. In 2015, the American College of Medical Genetics and Genomics (ACMGG) and the Association for Molecular Pathology (AMP) published the variant classification guidelines (Rehm et al., 2015), which significantly helped the variant classification process. However, the guidelines did not solve all of the problems, discordant results remain across laboratories (Amendola et al., 2016; Harrison et al., 2017). In China, exome sequencing technology has been adopted very rapidly. In addition to common issues, China is confronted with unique challenges: (1) a lack of professional, clinical, and medical geneticists, the equivalent of ABMGG-certified medical geneticists in the United States; (2) a lack of genetic counselors who play important roles in collecting/delineating clinical information, variant classification, report drafting, and genetic counseling for both physicians and patients; and (3) a lack of regulations like the Clinical Laboratory Improvement Amendments of 1988 (CLIA 88) in the United States. With these challenges in mind, the National Center of Clinical Laboratories (NCCLs) organized an initial assessment focusing on exome variant analysis and clinical reporting in China, as part of the effort to assess the performances of Chinese laboratories offering clinical diagnostic tests using exome. The assessment helped to identify issues related with the ability of laboratories to adapt to the ACMG/AMP guidelines. NCCLs also conducted a training program after the first survey and educated the laboratories on how to apply ACMG-AMP criteria correctly and move toward more consistent variant interpretations.

## Materials and Methods

### Raw Sequence Dataset

The initial survey involved three probands and two trios. The clinical manifestation and/or family history of these test cases suggested a possible genetic disease.

Genomic DNA was isolated from peripheral blood samples using the Gentra Puregene Blood Kit (Qiagen, Hilden, Germany) following the manufacturer’s protocol. The target regions for proband-only samples were captured by the ClearSeq Inherited Disease panel kit (cat No.5190–7519, Agilent Technologies, Santa Clara, CA). The whole exome for trio samples were captured by the SureSelect Human All Exon V6 kit (cat No.5190–8864, Agilent). Sequencing was performed on Hiseq X Ten (Illumina, San Diego, CA) using paired-end 150-bp reads according to the manufacturer’s protocol. Raw data (FASTQ files) were generated via the on-board Hiseq control software and reporter software (Illumina).

### Surveys and Analysis

Chinese laboratories that offer exome sequencing for routine clinical molecular testing were invited to participate in the survey. Participation was voluntary and free of charge. Participating laboratories downloaded the FASTQ datasets from a central portal. Laboratories were asked to process the data using their validated pipelines for alignment, variant calling, annotation and filtration, and variant interpretation and reporting as they would normally do for a clinical case. Participants were asked to provide a detailed description of the bioinformatic pipelines and quality metrics. In addition to the results of the variant classification, the laboratories were also asked to provide individual lines of evidence following the ACMG/AMP guidelines. The NCCLs collected all the results. The participants were asked to provide an explanation for missing identifying the causative variants.

After identifying the problems based on the initial assessment, a training program was designed and conducted, focusing on training the laboratories on how to apply the ACMG-AMP criteria correctly. The training included a one time in-person training course with workshops plus months of online educational lectures. After training, another five cases with putative disease-causing variants were sent to the same laboratories. The criteria applied by each laboratory were tracked to identify to what extent training improved the consistency and accuracy of variant interpretation.

## Results

### Clinical Findings and Genetic Variants in Survey Samples

The initial set (labeled as -1) and the second set (labeled as -2) of clinical cases are shown in Supplementary Table S1, which was provided to the participating laboratories along with the FASTQ files. Table 1 lists the phenotype-related variants in each case. All variants were confirmed by Sanger sequencing.

**TABLE 1:** Genetic variants in surveyed samples.

**Survey sample ID^*a*^**	**Intended variants and nomenclature**	**Variant type**	**Zygosity**	**Inheritance mode**	**Variant classification**	**Phenotype**
Survey 1–1811	NM_000441.1(SLC26A4):c.281C > T(p.Thr94Ile)	Missense	Het	AR	P	Deafness, autosomal recessive 4, with enlarged vestibular aqueduct(OMIM:600791)
	NM_000441.1(SLC26A4): c.919-2A > G	Splicing site	Het	AR	P	
Survey 1–1812	NM_000507.3(FBP1):c.720_729 delTTATGGGGCC(p.Tyr241Glyfs)	Frameshift	Het	AR	P	Fructose-1,6-bisphosphatase deficiency (OMIM:229700)
	NM_000507.3(FBP1): c.490G > A(p.Gly164Ser)	Missense	Het	AR	P	
Survey 1–1813	NM_000257.3(MYH7):c.2167C > G(p.Arg723Gly)	Missense	Het	AD	P	Cardiomyopathy, familial hypertrophic 1 (OMIM: 192600)
Survey 1–1814	NM_001854.3(COL11A1): c.3816 + 1G > A	Splicing site	Het	AD/*De novo*	P	Marshall syndrome (OMIM 154780)/ Stickler syndrome II (OMIM 604841)
Survey 1–1815	NM_017780.3(CHD7): c.1666-2A > G	Splicing site	Het	AD/paternal	LP	CHARGE syndrome(OMIM:214800)
Survey 2–1911	NM_006662.2 (SRCAP):c.7303C > T (p.Arg2435Ter)	Non-sense	Het	AD/*De novo*	P	Floating-Harbor syndrome (OMIM:136140)
Survey 2–1912	NM_000277.1(PAH):c.1197A > T (p.Val399 =)	Synonymous	Het	AR	P	Phenylketonuria (OMIM: 261600)
	NM_000277.1(PAH): c.728G > A (p.Arg243Gln)	Missense	Het	AR	P	
Survey 2–1913	NM_012123.4(MTO1):c.1291C > T(p.Arg431Trp)	Missense	Het	AR	LP	Combined oxidative phosphorylation deficiency 10 (OMIM: 614702)
	NM_0121233.4(MTO1):c.1390C > T(p.Arg464Cys)	Missense	Het	AR	LP	
Survey 2–1914	NM_000275 (OCA2):c.1503 + 5G > A	Missense	Het	AR	P	Albinism, oculocutaneous, type II (OMIM:203200)
	NM_000275 (OCA2):c.1441G > A (p.Ala481Thr)	Missense	Het	AR	VUS	
Survey 2–1915	IL2RG:c.664C > T(p.Arg222Cys)	Missense	Het	XLR	P	Severe combined immunodeficiency, X-linked (OMIM: 300400)

*Surveys 1–1811, 1812, and 1813 are proband-only raw data. Surveys 1–1814 and 1815 are trio raw data.*

### Participants and Performance

A total of 53 clinical laboratories from hospitals or commercial entities providing genetic testing participated in this survey. The necessary experience with WES data analysis was at least one year for these participates. At the deadline of the survey, 49 laboratories returned the results meeting the requirements. Each laboratory provided the variants associated with a phenotype, clinical diagnostic reports for each sample, and a brief description of their bioinformatics pipeline. Most participants (69.4%, 34/49) were from a commercial entity. As a result, 85.7% (42/49) of participating laboratories correctly identified all the variants related with a phenotype. A total of 42 laboratories reported the intended variant nomenclature as shown in Table 1. However, seven labs failed to identify eight disease-causing variants. One lab was a hospital laboratory, the other six were commercial laboratories. They subsequently provided the reasons for missing the variants which are summarized in Table 2. Two institutions (lab1 and lab2) identified the correct variants but reported the wrong ones due to clerical errors, exposing the quality control gap. One laboratory (lab3) missed the variants for sample 1815 due to a bug in the bioinformatics pipeline that resulted in the random termination of the annotation in dealing with trio data. The problem was fixed afterward. One institution (lab5) missed calling the indel variant in *FBP1*(NM_000507.3) c.720_729delTTATGGGGCC in sample 1815. Two institutions (lab4 and lab6) reported wrong variants by using the wrong phenotype. One participating laboratory (lab7) called a wrong indel variant (*FBP1* c.718_727delCCCCATAAGG).

**TABLE 2:** Summary of the reasons for wrong variants.

**Lab No.**	**Sample ID**	**Expected variant**	**Wrong variant reported**	**Reason for missing the right variant**
1	Survey 1–1814	*COL11A1* (NM_001854.3):c.3816 + 1G > A	*COL11A1* (NM_001854.3):c.4554 + 1G > C	Clerical error
2	Survey 1–1815	*CHD7* (NM_017780.3):c.1666-2A > G	*CHD7* (NM_017780.3:c.1666-2AA > G	Clerical error
3	Survey 1–1815	*CHD7*(NM_017780.3):c.1666-2A > G	False-negative	Bug in annotation script
4	Survey 1–1812	*FBP1(*NM_000507.3): c.490G > A/c.720_729delTTATGGGGCC	*ABCD1*(NM_000033):c.1489-6delC	Wrong filtration based on wrong phenotype capturing
5	Survey 1–1812	*FBP1(*NM_000507.3): c.490G > A/c.720_729delTTATGGGGCC	*NDUFAF6*(NM_152416):c.420 + 2_420 + 3insA	Not called by their bioinformatics pipeline
6	Survey 1–1815	*CHD7*(NM_017780.3):c.1666-2A > G	*COL11A2*(NM_080680.2):c.688G > T	Wrong filtration based on wrong phenotype capturing
7	Survey 1–1812	*FBP1(*NM_000507.3): c.720_729delTTATGGGGCC	*FBP1(*NM_000507.3): c.718_727delCCCCATAAGG	Wrong nomenclature for indel variant
8	Survey 1–1815	*CHD7*(NM_017780.3):c.1666-2A > G	*HSPG2*(NM_005529.6):c.4213G > C/c.9019G > A	Wrong inheritance mode

### Bioinformatics Pipeline Used by Participants

Generally, a bioinformatics pipeline consists of the following major steps: alignment, pre-variant call, variant calling, variant annotation, and filtration. The bioinformatics pipeline and its components used by participating laboratories for each step were highly variable as shown in Table 3.

**TABLE 3:** Bioinformatic pipeline elements used by participating laboratories.

**Bioinformatic pipeline steps**	**Tools used (%)**
Data quality control	FastQC (65%), in-house developed tools (16%), QualiMap (8%), Fastp (16%), Trimmomatic (18%), cutadapt (6%)
Sequence alignment	BWA-MEM (90%), SENTIEON (12%)
Pre-variant calling processing	Picard (57%), SAMtools (33%), GATK (69%)
Variant calling	GATK HaplotypeCaller (78%), SAMtools (10%), Freebayes (8%), SENTIEON (12%), DNAnexus (2%), Vardict (2%), Lofreg (2%)
Annotation	Annovar (82%), snpEff (10%), Ensembl VEP (12%), Annotools (2%), IVA (2%), and in-house developed tools (8%)
Pathogenicity prediction	dbscSNV (24%), SPIDEX (4%), GERP + + (6%), SIFT (39%), PolyPhen-2 (31%), LRT (8%), MutationTaster (16%), FATHMM (10%), MetaSVM (4%), MetaLR (14%), CADD (12%), InterVar (24%), REVEL (12%), M-CAP (8%), RadialSVM (4%)
Filtration	Annovar (49%), Phenolyzer (37%), Exomiser (33%), Phenomizer (4%), Ensembl VEP (2%), gemini (2%), Tgex (4%), Varseq (1%), in-house filtration (10%), IVA (2%)

The most used population frequency database was the 1000 Genomes project (94%), followed by ExAC (88%), gnomAD (63%), dbSNP (80%), ESP6500 (51%), and an in-house database (51%). A variety of knowledgebases and databases were used, including but not limited to: ClinVar (84%), OMIM (94%), Human Gene Mutation Database (HGMD) (84%), HPO (20%), CHPO (6%), COSMIC (16%), Orphanet (8%), an in-house annotation (22%), dbNSFP (69%), LOVD (14%), and DECIPHER (10%). A diverse range of pathogenicity prediction tools were utilized often in combinations of more than two.

### Comparison of Clinical Reports Between Two Surveys

Forty-five laboratories submitted clinical reports for each case in the first survey. The elements of the report are summarized in Table 4. It is notable that some laboratories did not clearly state the variants and associated information. Some important elements, such as tests performed, limitations of test methods, recommendations for genetic counseling, and further testing were reported by approximately only half of the laboratories (55.56%). A third of laboratories reported secondary findings following the ACMG 59 genes list (Kalia et al., 2017). Only two institutions indicated that they provide reanalysis of data after one year or after three months.

**TABLE 4:** Comparison of content for the clinical reports between the two surveys.

**Report elements**	**Percent of report (*n* = 45) in survey 1**		**Percent of report (*n* = 43) in survey 2**	
Patient name	44	97.78%	43	100%
Patient sex	45	100%	43	100%
Patient date of birth or age	45	100%	43	100%
Clinical symptoms and family history	34	75.6%	43	100%
Test indications	34	75.6%	43	100%
Specimen number	42	93.33%	43	100%
Date specimen collected	14	31.11%	30	69.77%
Date specimen received	36	80%	35	81.40%
Specimen type	42	93.33%	41	90.35%
Title	41	91.11%	43	100%
Laboratory contact information	25	55.56%	43	100%
Ordering physician	31	68.89%	40	93.02%
Signatures	38	84.44%	43	100%
Date of report issued	40	88.89%	43	100%
Current/total page	35	77.78%	40	93.02%
Test performed (e.g., WES/panel/WGS)	28	62.22%	42	62.22%
**Description of test method**	13	28.89%	27	62.79%
Sequencing instrument and sequencing information	11	24.44%	26	60.47%
Description of bioinformatics pipeline	14	31.11%	14	32.56
Technical parameters (e.g., mean depth of coverage across the target region, coverage on target region)	18	40%	24	55.81%
Limitations and validity of test method	35	77.78%	42	97.67%
**Test results**	45	**100%**	43	100%
Human reference genome build (e.g., UCSC hg19)	21	46.67%	28	65.12%
Chromosome number and gene coordinates	36	80%	36	90.70%
Gene name	45	100%	43	100%
Exon or intron number	25	55.56%	34	79.07%
Transcript	43	95.56%	43	100%
Nomenclature at both the nucleotide (genomic and cDNA) and protein level using HGVS nomenclature	45	100%	43	100%
Zygosity	44	97.78%	43	100%
Variant classification (pathogenic)	45	100%	43	100%
Mode of inheritance (AD, AR)	43	95.56%	41	95.35%
Parental inheritance	38	84.44%	38	88.37%
A summarized conclusion	27	60%	33	76.74%
**Test interpretation**	40	88.89%	35	90.70%
Recommendations for genetic counseling and further testing	25	55.56%	38	88.37%
References	35	77.8%	32	74.42%
Statement of laboratory’s availability	20	44.4%	24	55.81%

A training meeting was subsequently conducted in order to standardize the content and format of clinical reports. The reports submitted for the second round of surveys had a much improved consistency as shown in Table 4.

### Performance and Improvement of ACMG-AMP Variant Classification

**FIGURE 1:**
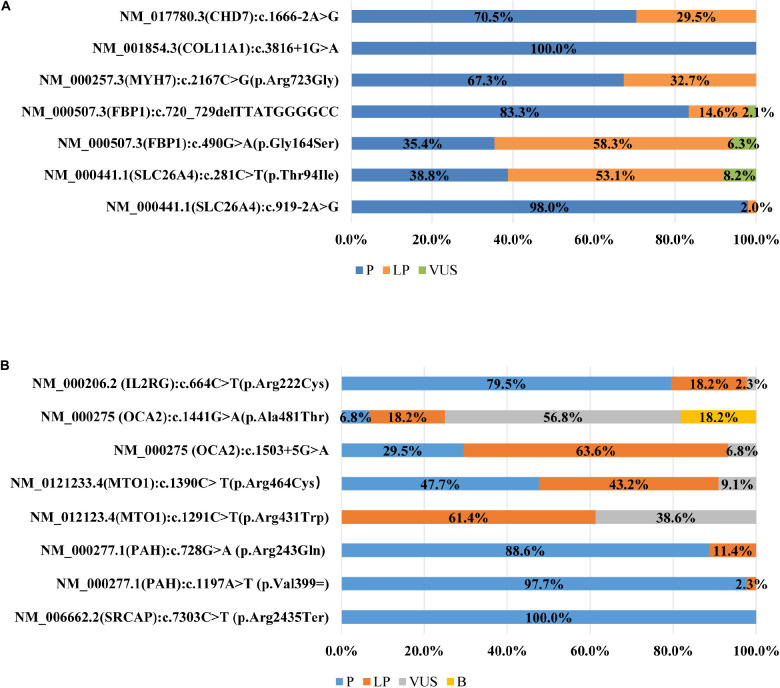
**(A)** Distribution of variant classification in the first survey (*n* = 45). **(B)** Distribution of variant classification in the second survey (*n* = 43).

All participating laboratories claimed that they assessed the pathogenicity of variants following the ACMG/AMP guidelines (Richards et al., 2015), but the outcomes were quite different, and the concordance rate differed greatly from variant to variant. Of the seven variants (Figure 1A) in the first survey, the classification of four variants were concordant across laboratories, with only one variant in exact agreement and three variants only differing in P versus LP. The classifications of the remaining three variants (*FBP1*:c.490G > A, *SLC26A4:*c.281C > T, and c.720_729del) were quite discordant, the classification crossed clinical categories (i.e., P/LP versus VUS). For 3.3% (11/335) of the variant assessments, the ACMG evidence listed by the laboratories did not support the classification chosen.

**FIGURE 2:**
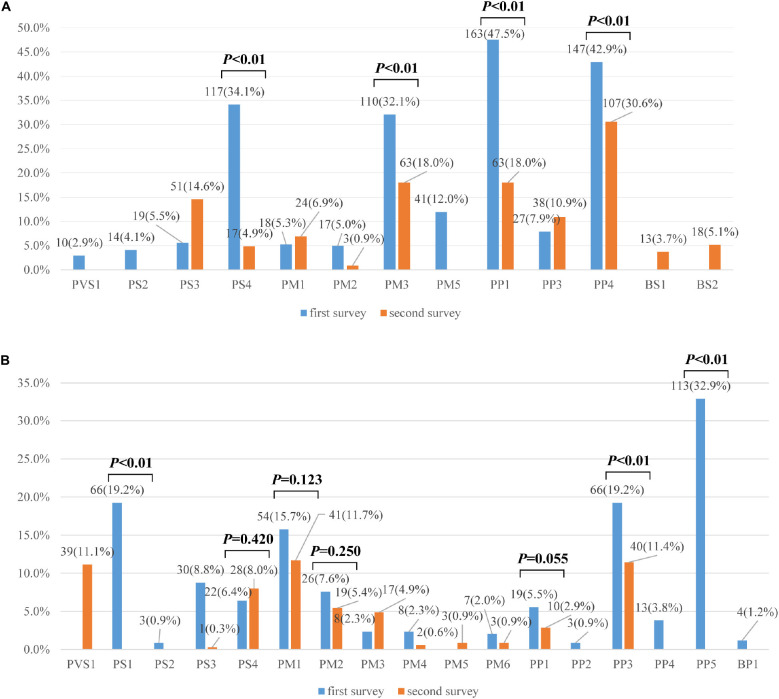
**(A)** Percentages of category 1 errors across lines of evidence between the first and second surveys (*n* = 335). **(B)** Percentages of category 2 errors across lines of evidence between the first and second surveys (*n* = 335). Legend: Lines of evidence that should have been used but were not are category 1 errors, those that should not have been used but were are category 2 errors.

A total of 16 different lines of evidence were invoked for variant classifications in the survey. PM2 (absent from controls), PP3 (*in silico* evidence), and PVS1 (variant predicted null where loss of function is a mechanism of disease) were the most frequently used lines of evidence in the first survey. Lines of evidence that should have been used but were not are plotted in Figure 2A **(category 1 error) and** those that should not have been used but were are plotted in Figure 2B **(category 2 error).** Category 1 error occurred in over 30% of instances for some lines of evidence. PP1 (segregation data) (47.52%), PM3 (allelic variant in-trans) (32.07%), and PS4 (case-control difference) (34.11%) were among the most significantly misused lines of evidence as part of category 1 error. PP4 (using phenotype to support variant claims) (42.86%) was also the most inconsistently used evidence among the laboratories. Category 2 error occurred in over 15% of instances for some lines of evidence. PP5 (reputable source) was still used for variant classifications (32.94%), though ACMG-AMP had proposed that laboratories discontinue the use of this criteria. PS1 (same amino acid change) (19.24%), PP3 (functional prediction) (19.24%), PM1 (mutational hot spot and/or critical and well-established functional domain) (19.24%) were among the most significantly misused lines of evidence as part of category 2 error. The misusage of PP3 was due to ‘double counting’ when PVS1 had already been used. Some laboratories used PS1 when the same missense variant had been reported before. Some used PS1 instead of the PM5 (novel missense at the same position) criteria for the MYH7 c.2167C > G (p. Arg723Gly) variant. The misusage of PM1 was due to a misunderstanding of the well-established functional domain. Only three laboratories correctly up- and downgraded lines of evidence (up-graded for PM3 and PP1 and down-graded for PM3, PS4, and PS3).

In the second survey, three of eight variants had consistent classifications across laboratories (Figure 1B). While the classification for the other five variants were still discordant, the ACMG evidence codes listed by the institutions all supported their chosen classification and the overall error rates for some lines of evidence were greatly reduced compared to the first survey (Figures 2A,B). For example, the percentage of category 1 error dropped from 47.52%to 18.00% for PP1, from 32.07% to 18.0% for PM3, and from 34.11% to 4.86% for PS4. Yet there was still a high error rate for PP4 (30.57%). The rates for category 2 error dropped to 0 for both PS1 and PP5, and dropped from 19.24% to 11.43% for PP3. The laboratories were still relatively confused on how to use PM1. PVS1 (Null variants) (11.14%) was also a significantly misused line of evidence as part of category 2 error, this is because the disease mechanism can be difficult for *SRCAP* mutations. More laboratories modified the strengths of evidence to make it more appropriate for certain lines of evidence (four lines of evidence - PS2, PM3, PP1, and PP4 were upgraded and three lines of evidence - PM3, PS4, and PS3 were downgraded.

## Discussion

In 2015, ACMG and AMP published the guidelines for variant classification (Richards et al., 2015). In 2017, the Chinese translation of the guideline was published and it was recommended that all Chinese clinical laboratories adopt the guidelines for clinical diagnostic services (Wang et al., 2017). However, we knew very little about how well the guidelines were followed and most importantly, whether the guidelines were used correctly in actual practice across different laboratories in China. These concerns are particularly pressing since we do not have a well-established professional training and quality assurance program for molecular diagnostic testing in China, yet the volume of clinical diagnostic tests is the largest in the world due to a large patient population and the relatively low cost of testing in China (Hu et al., 2018). For this reason, we conducted this survey to identify problems in our current practice including issues with the secondary data analysis, variant classification, and clinical reporting.

### The Secondary Analysis of Exome Data

There is no uniform or recommended bioinformatics pipeline for clinical exome testing in China. Most laboratories developed their own pipeline by combining different open-source algorithms or commercial software for secondary analysis of exome data. Thus, it is difficult to perform a detailed and direct comparison between laboratories. Instead, we looked for the final reportable variants. As a result, the survey exposed several errors due to the bioinformatics pipeline used, the errors included wrong variant calling, wrong variant annotation, and incorrect filtering. In addition, even for laboratories using similar software components, there was no consensus on quality control (QC) metrics. The summary of quality metrics provided by participating institutions are provided in Supplementary Figure S1. Developing a robust and cohesive diagnostic pipeline to achieve optimal NGS testing quality is still a challenge for some Chinese diagnosis laboratories. The problems were rooted in the fact that there was a lack of vigorous bioinformatics pipeline validation before the required implementation of standards and guidelines for routine use (Rehm et al., 2013; Hegde et al., 2017; Roy et al., 2018). Ultimately, the lack of validation was due to the lack of accreditation for clinical laboratories and certification for personnel, which in turn should be overseen by a regulatory body like CLIA. Our findings indicated that implementing routine proficiency testing for the bioinformatics portion of the NGS assay is necessary in China. Several corrective actions for the improvement of the secondary analysis pipeline could be implemented based on specific issues exposed during proficiency testing. A future proficiency test could consider sending raw data with VUS or negative results to laboratories to see whether the laboratories correctly identify VUS or negative results, and consider sending repeated reference material such as NA12878 and other clinical samples so that participating laboratories submit raw datasets (i.e., BAM and VCF). Detailed inter-laboratories data quality parameters such as coverage consistency and precision of variant calling would be informative for the continued standardization and improvement of the secondary analysis of exome data. There will be insights into what the impact of using different tools is and whether the discrepancies were related to software or whether it was due to inexperience in the application of these tools (i.e., setting thresholds for variant calling and FPs). If it is necessary, training entailing information about bioinformatics pipeline calling could also be performed in the future.

### Variant Classification

It has been a major challenge to accurately and consistently classify variant pathogenicity. The ACMG/AMP guidelines provided a great framework for variant clinical interpretation and helped to significantly reduce discrepancies (Amendola et al., 2016; Garber et al., 2016; Niehaus et al., 2019). But the implementation of the guidelines does not eliminate the issues. For example, even with a consensus effort of using the ACMG guidelines, 21% and 29% of variants showed intra- and inter-laboratory discordance, respectively (Amendola et al., 2016). Similar discordance rates (22–25%) were reported by Garbor et al. and 28% of variants remained discordant after a harmonization effort (Garber et al., 2016). The main purpose of our survey was to identify the problems associated with the proper use of the lines of evidence for variant classification following the ACMG/AMP guidelines in China. Since only a limited number of variants were assessed by the laboratories, the findings are qualitative rather than quantitative. Garbor et al. pointed out that the discrepancies were mainly due to the time difference between when the variant was assessed, the availability of internal data, and the use of different AF cutoffs. While these are important factors affecting the accuracy of variant classification, the problems in China, as this survey found, are more rudimentary. Many discrepancies were due to the incomplete understanding or misunderstanding of the meaning of the evidence. For example, the ACMG evidence codes listed by eight institutions did not support their chosen classification for some variants, due to inexperience in using the rules for combining criteria to classify sequence variants. PS1 was often used when the exact variant was previously reported as pathogenic. PP3 was used when PVS1 was already invoked. Many laboratories did not keep up with guideline updates so PP5 continued to be used, up and downregulations were not practiced when the information was available. The findings of our initial survey underscore the importance of training for personnel in Chinese clinical laboratories. Significant reductions in both category 1 and 2 errors in evidence utilization after the training supported the benefit of such activity conducted by the NCCLs. But there are still other issues: 1) though ClinGen’s Sequence Variant Interpretation Expert Panels are publishing specifications of the guidelines to further clarify how certain criteria are applicable for a given gene or disease, some Chinese laboratories do not pay enough attention to updates and do not apply them for variant interpretation for a given gene or disease. For example, the ClinGen PAH Expert Panel published specifications to the ACMG/AMP variant interpretation guidelines for the PAH gene^1^. It specified that the *in vitro* enzyme activity < 50% compared to wild-type control or RT-PCR evidence of mis-splicing could be used as PS3 (functional evidence). However, PS3 was not used by 13.6% (6/44) of laboratories in variant classifications for the PAH gene in the second survey. Also, there was a lot of uncertainty associated with the use of PS3. It was not used in variant classifications for 14.57% of variants in the second survey, illustrating that the evidence is still challenging for many laboratories; 2) up and downgrading evidence became the main cause of discordance for lines such as PP1, PM3, and PS2/PM6. Many laboratories had not implemented the upgraded guidelines or had difficulty in judging when it was appropriate to up or downgrade the evidence, or literature searching was incomplete so some raw evidence was missed. On the other hand, we trained laboratories on how to interpret the loss of function PVS1 in the ACMG/AMP variant criterion (Abou Tayoun et al., 2018). In a future survey, we will examine the correct usage of PVS1 in upgrading and downgrading evidence; 3) in China, genetic tests are not only offered by a few hospital laboratories who have clinical and research experience with genetic disorders, but also by laboratories or commercial entities without an extensive knowledge about genetic disorders. Also given the fact that most ordering physicians were not well-trained in medical genetics, the patients did not receive a thorough phenotypic evaluation and differential diagnosis, and thus the orders were often accompanied with very limited clinical information. Consequently, many laboratories were not able to properly use PP4, either due to unavailable insufficient information or the incapability of physicians in performing clinical assessment. For example, in this survey, about half of the laboratories (19/44) did not use PP4 evidence when we provided sufficient clinical information that were well-matched with the phenotype of Floating-Harbor syndrome (FHS). In addition, there was a lack of effective interaction with the ordering physician, so when the phenotypic information was incomplete, the lab would miss the variant. This is the case for the lab that missed the “obvious” variant *CHD7*:NM_017780.3, c.1666-2A > G, because they thought the provided information was the “whole observable phenotype”, and assumed that the patient did not have any other abnormalities. The lack of personnel with clinical experience will be a significant problem for genetic testing in China if a certification program is not implemented. It should be noted that the number and type of variants in the surveys were limited and not all relevant issues had been identified by this initial assessment. But improving the understanding of the guidelines can help with certain issues right away. Our near-future efforts will focus on further training for personnel and the development of additional practice guidelines to assist with the application of the ACMG-AMP guidelines in Chinese laboratories. During the training we will illustrate the importance of data and experience sharing, NCCLs will organize a clinical consortium to facilitate fair data sharing and resolve inter-laboratory discrepancies, as was already implemented by ClinGen (Harrison et al., 2017). In the future survey, a wider variety of variants including benign variants will be distributed to more laboratories by NCCLs, this effort will help to clarify common usage errors and identify challenges in Chinese genetic laboratories.

### Clinical Reporting

Although specific international guidelines (Aziz et al., 2015; Richards et al., 2015) and Chinese standards (Huang et al., 2018) have been published and recommended for clinical reporting, our survey also identified many issues with the reports. It was noted that most clinical reporting did not include all the essential information on performed NGS-based testing, including description of the test method, limitations, the validity of the test method, description of genetic counseling, recommendations for next steps, and the human reference genome build (as shown in Table 4). Our training and the feedback of the survey results to the participating laboratories emphasized the essential elements of the report and assisted the participating laboratories in helping them to continuously improve the format and content of their clinical reports. Our work has been useful in helping laboratories develop a clinical report process with the clinical significance on any relevant findings clearly and concisely stated and comprehensible to clinicians.

The overall purpose of this initial survey in China was to investigate the performance of exome variant analysis and clinical reporting and to identify the shortcomings of the process and its product. We have used training as one of the solutions to amend the identified problems. We also implemented several corrective actions based on the specific issues exposed during the surveys including the adjustment and improvement of the secondary analysis pipeline, the implementation of appropriate validation, and a more vigorous process of variant interpretation and reporting. In conclusion, the practice of NGS-based exome sequencing for genetic disease in China is still in the early stages of its development. The initial survey revealed a significant number of issues associated with but not unique to the clinical exome test in Chinese laboratories. We also illustrated the approaches that can help to achieve optimal exome testing quality in Chinese diagnostic laboratories.

## Data Availability Statement

All datasets presented in this study are included in the article/Supplementary Material.

## Author Contributions

KZ drafted the manuscript. YS and JL revised the manuscript. JW, DH, YH, and GL contributed to the acquisition and interpretation of data. KZ, YS, and JL designed the work. All authors contributed to the manuscript revision, read, and approved the submitted version.

## Conflict of Interest

The authors declare that the research was conducted in the absence of any commercial or financial relationships that could be construed as a potential conflict of interest.

## Footnotes

1 https://www.clinicalgenome.org/affiliation/50015
