# Supplementary figures and images for: An Initial Survey of the Performances of Exome Variant Analysis and Clinical Reporting Among Diagnostic Laboratories in China
Source: Front Genet. 2020 Nov 2;11:582637. doi: 10.3389/fgene.2020.582637 (PMC7667017; doi:10.3389/fgene.2020.582637)

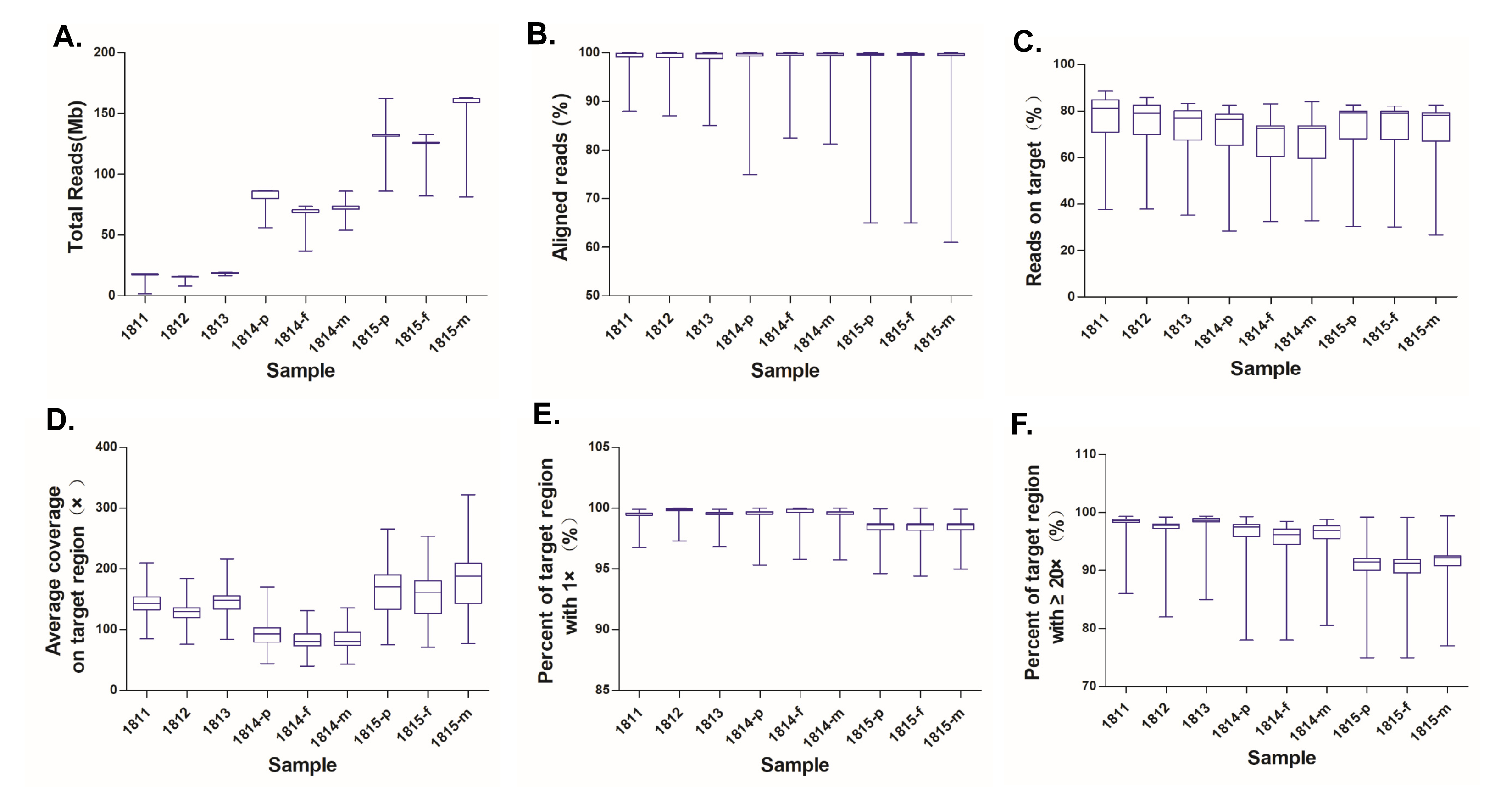

Supplement: Supplementary file 1 [file Image_1.TIF]
