# Supplementary material for: An Initial Survey of the Performances of Exome Variant Analysis and Clinical Reporting Among Diagnostic Laboratories in China
Source: Front Genet. 2020 Nov 2;11:582637. doi: 10.3389/fgene.2020.582637 (PMC7667017; doi:10.3389/fgene.2020.582637)
Supplement: Supplementary file 2 [file Table_1.DOCX]

**Table S1 Clinical findings in survey samples**

| Sample ID^a^ | Phenotype | Clinical history and information |
| --- | --- | --- |
| Survey 1-1811 | Deafness, autosomal recessive 4, with enlarged vestibular aqueduct（OMIM:600791） | A 22-year-old female was diagnosed with congenital bilateral hearing loss since birth.  MRI confirmed the enlarged endolymphatic duct and sac.  No special treatment was given.  No family history. |
| Survey 1-1812 | Fructose-1,6-bisphosphatase deficiency（OMIM:229700） | Proband was an 11-year-old male with history of recurrent attacks of epilepsy associated with vomiting, drowsiness, or tachypnea.  He experienced attacks of hyperventilation while weaning at the age of 10 months.  Laboratory studies showed hypoglycemia and severe metabolic acidosis, lactic acidosis.  Therapeutic measures included oral administration of sodium valproate and Topamax.  No family history of epilepsy. |
| Survey 1-1813 | Cardiomyopathy, familial hypertrophic 1 (OMIM: 192600) | Proband was a 4-month-old female with history of tachypnea, feeding difficulties, and severe pneumonia.  She was born premature and had a history of infection and respiratory failure at NICU.  Mother had hypertrophic nonobstructive cardiomyopathy.  MRI confirmed the left ventricular septa and parts of ventricular wall were thickened. There was a right ventricular septal defect. Electrocardiogram showed nodal tachycardia. |
| Survey 1-1814 | Marshall syndrome  (OMIM 154780)/  Stickler syndrome II  (OMIM 604841) | Proband, a 5-year-old female, was noted with short stature and unusual face and was referred to endocrine clinic.  The proband was found to have high myopia as well as moderate hearing loss at the age of 3.  She presented with midface hypoplasia, short nose with anteverted nares.  No affected family members. |
| Survey 1-1815 | CHARGE syndrome（OMIM：214800） | The baby was a product of G1P1 with abnormal prenatal ultrasound findings. His father had orofacial cleft and moderate hearing loss of right ear. The baby presented with congenital heart defects, external ear defects and hearing loss, pulmonary arterial hypertension. He died soon after birth. |
| Survey 2-1911 | Floating-Harbor syndrome (OMIM:136140) | Proband was a 6-year-old male and was noted with short stature and delayed bone age (<–2 SD). He also exhibited with expressive language delay, and a triangular face with a prominent nose, deep-set eyes, a wide columella, and thin lips. Both parents had normal stature. The family history was negative. |
| Survey 2-1912 | Phenylketonuria (OMIM: 261600) | Proband, a 6-month-old male, had found intellectual disability with mousy odor and light pigmentation.  The plasma phenylalanine concentrations were persistently above 120μmol/L (2mg/dL), and the urine pterins and DHPR activities were normal.  There was no family history. Parents were non-consanguineous. |
| Survey 2-1913 | Combined oxidative phosphorylation deficiency 10（OMIM: 614702） | Proband, a 5-month-old male, presented with poor feeding, hyperpnea, hypotonia and severe metabolic acidosis soon after birth. Ultrasound examination revealed hypertrophy of the septum and left ventricular wall.  The proband had two brothers, both died at infancy. One with cardiomyopathy and lactic acidosis.  Another with severe metabolic acidosis at birth with multiple organ failure and severe pneumonia. WES was performed on the two sibs and identified the same compound heterozygous variants as the proband had. |
| Survey 2-1914 | Albinism, oculocutaneous, type II (OMIM :203200) | Proband was a 2-year-old female with light skin, hair and eyes. She showed impaired vision and photophobia.  No other affected family members. |
| Survey 2-1915 | Severe combined immunodeficiency, X-linked (OMIM: 300400) | Proband, a 10-month-old male, G3P3, presented with diarrhea. gamma globulin administration showed no effect and he died at the age of 11 months.  The first child of the couple had recurrent bronchitis, died of severe dehydration. The second child had infection before 3 months of age, followed by lymphopenia and persistent pneumonitis, moniliasis and frequent rashes. He died at 7 months of age.  WES testing was performed on the blood samples of the second child and the same variant was detected as the proband. |

**Supplemental Figure S1; The summary of QC metrics from participating laboratories（n=49）**
